# “You matter”: patients perceptions and disparities about  cancer care and telehealth during and after COVID-19 pandemic

**DOI:** 10.1007/s00520-024-08433-2

**Published:** 2024-03-20

**Authors:** Mohamed Mohanna, María Herrán, Barbara Dominguez, Saad Sabbagh, Ali Msheik, Mira Itani, Ludovic Saba, Sindu Iska, Hong Liang, Caroline Metzel Diaz, Zeina Nahleh

**Affiliations:** 1 Department of Hematology and Oncology, Cleveland Clinic Florida, 2950 Cleveland Clinic Blvd, Weston, FL 33331 USA; 2 Department of Neurosurgery, Faculty of Medical Sciences, Lebanese University, Beirut, Lebanon; 3 Department of Family Medicine, Emory, Atlanta, Ga USA; 4 Department of Hematology and Oncology, University of Arkansas Medical Sciences, Little Rock, AR USA; 5 Department of Research, Cleveland Clinic Florida, Weston, USA

**Keywords:** Telehealth, Cancer care, COVID-19, Telemedicine

## Abstract

**Purpose:**

Disparities in cancer care have been exacerbated by the COVID-19 pandemic. The aim of this study is to establish how telehealth mitigated the effect of COVID-19 on the healthcare sector and to identify potential disparities in perception and experience with telehealth in cancer care during and after the pandemic.

**Methods:**

We identified individuals with an established cancer diagnosis who received treatment at a comprehensive academic cancer center with a diverse patient population between 2019 and 2021, during the COVID-19 pandemic. Participants were asked to complete a self-administrated survey intended to collect patient-reported outcomes on socioeconomic and mental health challenges incurred during the pandemic as well as participants’ experience with telehealth. The assessment was adapted from a 21-question-based survey applied for mental health. Descriptive statistics were used to summarize participant characteristics and the response to the survey items. Multivariable logistic regression was performed to assess and analyze the contributing factors to the survey responses.

**Results:**

A total of *N* = 136 participants were included in this analysis. The majority of participants (60.6%) reported increased anxiety, stress, or experience of distress as a direct result of COVID-19. However, among 54.1% of survey responders participated in a telehealth appointment and 84.4% agreed it was an easy and effective experience.

**Conclusion:**

Elderly, male, and black participants reported the worst impact related to the pandemic. The majority of patients had a positive experience with telehealth. The results of the study suggest that telehealth services can serve as a tool for patients with cancer during and beyond active treatment to access supportive services.

**Supplementary Information:**

The online version contains supplementary material available at 10.1007/s00520-024-08433-2.

## Introduction

There has been major concern regarding the impact of COVID-19 on essential health services, particularly cancer management. Patients with cancer experienced pandemic-related delayed appointments, canceled screening tests, or unexpected changes in treatment plans, among others [1–3]. Nevertheless, cancer management is extremely time-sensitive, and delays in treatment plans may lead to unintended repercussions. Therefore, healthcare professionals pivoted to provide medical services remotely to ensure continuity of care and reduce COVID-19 exposure [4].

According to the American Telemedicine Association, the term *telemedicine* is used to characterize the healthcare services delivered by electronic methods to enhance the health of an individual, whereas *telehealth* involves clinical services, administration, education, and research [5]. However, these two terms have been commonly employed interchangeably in the literature to describe the use of technological communication capabilities to deliver medical care remotely [6]. Potential benefits of telehealth have been reported, including access to clinical services for patients in remote locations, reduced healthcare costs, provision of previously unavailable care, and enhanced professional education [7].

Consequently, the rapid implementation of telehealth during COVID-19 has resulted in a paradigm shift in oncology services, in which digital services have become an effective norm for healthcare delivery [8]. Even though multiple studies have suggested that patients with cancer have generally responded favorably to telemedicine visits [9–11], published data lacks the acceptance of telehealth among patients. Furthermore, we have yet to standardize the use of telehealth in providing patients with supportive services, including mental health assessments and therapies.

This is particularly relevant in cancer care since, compared to the general population, patients with cancer are at a higher risk of developing mental health disorders, with only a small percentage of patients seeking psychological counseling [12]. This could worsen outcomes since psychological discomfort in cancer patients has been reported to lead to a negative impact on their physical health, treatment adherence, and quality of life [13, 14]. COVID-19 may have exacerbated the burden of cancer, and it is crucial that we identify the factors associated with the increasing prevalence of such mental health concerns [15, 16].

In this analysis, we conducted a patient-reported questionnaire at a comprehensive academic cancer center after COVID-19 to evaluate changes in patients’ psychosocial health and attitudes toward telehealth visits. Our aim is to determine if an association exists between patients’ socio-demographic attributes and patient-reported outcomes. Understanding patients’ experience with telehealth and defining the factors that may restrict patients from using telehealth and access to mental health services were important objectives of this study. Telemedicine will likely continue to be a reliable resource for providers, especially for integrative oncology, in the post-pandemic era. Therefore, it would be helpful to evaluate the long-term efficacy and the acceptance of telehealth as part of the standard of care in promoting a better quality of life while reducing distress and psychosocial morbidity associated with cancer [17].

## Methods

### Participants and procedures

Patients diagnosed with cancer at the Maroone Cancer Center, Cleveland Clinic Florida, who met the inclusion and exclusion criteria were invited to participate in a brief cross-sectional survey from November to February 2022. Inclusion criteria included (1) age 18 years or older; (2) any cancer diagnosis at any pathologic stage or histology; (3) established patients followed by a medical oncologist, radiation oncologist, and/or surgeon at our center; and (4) able and willing to participate in the survey.

### Questionnaire development and participant recruitment

This is a non-interventional survey-based evaluation of patient-reported perception of telehealth cancer care and access to digital services. The survey is based on the “You Matter: Covid-19 Mental Health Impact Survey” by the Connecticut Department of Mental Health and Addiction Services (CT DMHAS) [18]. We then adapted and refined the 21-item survey into four main categories based on the objectives of this study: demographics, social health, telehealth attitudes, and mental health (Supplemental Table). Survey questions were multiple choice—some eliciting answers on a scale of “yes,” “no,” and “I don’t know” and others eliciting answers on a 5-point Likert scale. Moreover, patients attending the outpatient clinic were provided with a survey, and responses were captured in an electronic and secure database. Patients had the option to leave a question blank.

#### Social health

Using a 5-point time-anchored scale, participants were asked to rate whether they perceived a change (much worse, somewhat worse, about the same, somewhat better, and much better) in their overall health, physical and mental health since April 2020. We also sought to ask if patients had health insurance, if there was any change in their coverage, the number of household members and level of stress, and if they experienced any challenges during the pandemic. Challenges were defined as any difficulty involving housing, income, food, toiletries, transportation, education, healthcare, or mental health care.

#### Telehealth attitudes

A total of six questions were included in the telehealth attitudes section and distributed in the following domains: participation in any telehealth appointment, mode of communication, difficulty level using telehealth, contact with providers, obstacles encountered when using digital services, and comfort level with telehealth.

#### Mental health

This section of the survey consisted of three questions; participants were asked about their perception of their mental health, in-person health or physical health appointments since April 2020, and level of difficulty receiving mental health or substance use services.

### Ethical considerations

The study was conducted in compliance with the International Council for Harmonisation (ICH) guidelines and with all applicable federal (including 21 CFR parts 56 and 50), state, and local laws. No protected health information (PHI) was collected in this study, and all anonymized survey results were captured via RedCap.

### Statistical analysis

Consecutive established patients with cancer were identified from the cancer center database and enrolled in this study. Descriptive statistics, including frequency number and percentage, were used to summarize participant characteristics and responses to the survey items. For the primary objective, univariate analysis was performed to assess the association of variables with patients’ characteristics, including age, gender, race, education, insurance, marital status, employment, comorbidity, cancer stage, treatment status, and patients’ performance status. In addition, multivariable logistic regression analysis with the backward elimination method was performed to identify associative variables contributing to the outcomes. These variables were included in the multivariable logistic regression model as explanatory variables. Similar methods were used to analyze other outcomes. All data analysis was conducted using SPSS version 28 or SAS version 9.4.

## Results

A total of *n* = 136 consecutive patients completed the survey and were included in this study. Most participants were females (70.4%), white (75%), and had an age ≥ 65 (70.4%). The cancer center serves an area with relatively high Hispanic prevalence, as represented in the sample with 31 patients (22.8%) reporting Hispanic ethnicity. Less than a half of the patients reported having a bachelor’s or higher degree (43%); the majority had > 1 household member (80.1%), were not covered by private insurance (62.5%), and did not change insurance during pandemic (94.9%). The detailed demographic features of participants are shown in Table 1.

**Table 1:** Characteristics of patients who completed the Patient-Reported Outcomes (PRO) Survey

Survey item	*N* (%)
Demographics	
Gender	
Male	40 (29.6)
Female	95 (70.4)
Age	
34–65	40 (29.6)
≥ 65	95 (70.4)
Race	
White	102 (75.0)
Black	23 (16.9)
Other	11 (8.1)
Hispanic	31 (22.8)
Education	
High school or lower	22 (16.3)
Some college	55 (40.7)
Bachelor’s or higher	58 (43.0)
Social health	
Insurance	
Private	51 (37.5)
Other	85 (62.5)
Insurance change during the pandemic	
No	129 (94.9)
Yes	7 (5.1)
Experienced challenges^a^ because of COVID-19	
No	75 (55.1)
At least one	61 (44.9)
Stress increase	
No	69 (50.7)
Yes	67 (49.3)
Number of people in the household	
1	27 (19.9)
> 1	109 (80.1)
Overall health	
Worse	42 (30.9)
Not worse	94 (69.1)
Physical health	
Worse	46 (33.8)
Not worse	92 (66.2)
Mental health	
Worse	27 (19.9)
Not worse	109 (81.1)
Telehealth attitudes	
Have you participated in tele-appointments since April 2020?	
No	62 (45.9)
Yes	73 (54.1)
Experience with telehealth	
Easy/effective	65 (84.4)
Difficult	12 (15.6)
Contact with provider since April 2020	
Less contact	14 (10.3)
More contact	122 (89.7)
Comfortable with telehealth	
Not comfortable	35 (29.4)
Comfortable/neutral	84 (70.6)
Mental health	
Received mental/physical health service since April 2020	
Yes	62 (46.3)
No	72 (53.7)
Experience an increase in anxiety, stress, or distress as a result of COVID-19	
Increase (slight, moderate or significant)	80 (60.6)
Not increase	52 (39.4)
Difficulty in receiving mental health or substance use services	
Difficult/somewhat difficult	12 (34.3)
Not difficult	23 (65.7)

^a^Includes housing, income, food, toiletries, transportation, education, healthcare, or mental health care

Regarding telehealth attitudes, more than half of the patients reported participating in tele-appointments since April 2020. Among this group, the majority expressed comfort (70.6%) and described their telehealth experience as easy and effective (84.4%), and 89.7% reported that the frequency of digital contact with the provider increased after implementing digital strategies during the COVID-19 pandemic. However, in the multivariate analysis (Table 2), we found that participants 65 years or older were more likely to express discomfort with telehealth compared to younger participants (OR 3.1; 95% CI 1.14, 8.45; *p* = 0.02), and this phenomenon was less frequent in males than in female participants (OR 0.034; 95% CI 0.12, 0.93; *p* = 0.035). Black participants were > 4 times more likely to report less contact with their physician (OR 5.54; 95% CI 1.31, 23.52; *p* = 0.018).

**Table 2:** Multivariable logistic regression analysis for the survey responding events

	*N* (%)	OR (95%CI)	*p*-value
Experienced at least one challenge^*a*^			
Gender			
Female	37 (39.0)	1	**0.0496**†
Male	23 (57.5)	2.12 (1.00–4.49)	
Worse at overall health			
Age			
< 65	7 (17.5)	1	**0.0359**†
≥ 65	34 (35.8)	2.67 (1.07–6.69)	
Less contact with provider			
Race			
White	6 (5.9)	1	**0.0180**† 0.3490
Black	6 (26.1)	5.54 (1.31–23.52)	
Other	2 (18.2)	3.50 (0.30–23.79)	
Not comfortable with telehealth			
Age			
< 65	6 (16.2)	1	**0.0210**†
≥ 65	29 (35.8)	3.10 (1.14–8.45)	
Gender			
Female	29 (35.4)	1	**0.0350**†
Male	6 (16.7)	0.034 (0.12–0.93)	
Increased anxiety, stress, or experience of distress as a result of COVID-19 “Mental health”			
Gender			
Female	60 (65.9)	1	**0.0490**†
Male	19 (47.5)	0.47 (0.22–1.00)	
Received mental health services			
Race			
White	51 (51.0)	1	0.9653 0.0007
Black	11 (47.8)	0.88 (0.32–2.41)	
Other	0 (0.0)	0.07 (0.00–0.32)	
Education			
Bachelor’s degree or higher education	34 (58.6)	1	0.0507 0.0249
High school education	7 (31.8)	0.33 (0.11–1.00)	
Any college education	21 (39.6)	0.39 (0.17–0.89)	
Hispanic			
No	55(52.9)	1	0.0012
Yes	7(23.3)	0.19(0.07–0.51)	
Insurance			
Private	27(54.0)	1	0.0497
Other	35(41.7)	0.45(0.20–1.00)	

†Statistically significant

^a^Challenges were defined as any difficulty involving housing, income, food, toiletries, transportation, education, healthcare, or mental health care

When evaluating perceptions about mental health, more than half of the participants reported an increase in anxiety and distress (60.6%). Furthermore, males were less likely to experience these symptoms than female patients (OR 0.47; 95% CI 0.22, 1.00; *p* = 0.049).

In the social health domain of the survey, 44.9% of the participants experienced at least one challenge during the pandemic, and males were more likely to experience challenges (OR = 4.49, 95% CI 1.00, *p* = 0.049). Only 30.9% of the respondents were considered to have an exacerbation in overall health, and participants aged ≥ 65 were more likely to report this outcome compared to younger participants (OR 2.67; 95% CI 1.07, 6.69; *p* = 0.0359).

## Discussion

This analysis confirms the negative impact of COVID-19 on patients with cancer overall and highlights significant differences in outcomes based on age, gender, and race. This is consistent with other reports suggesting an increased rate of psychological distress since the pandemic [19]. This analysis also elucidated the utilization and attitudes toward telehealth for cancer care and suggested that the majority of patients with cancer express readiness and a positive experience with telehealth service, but older patients and males noted less comfort than younger patients < 65 years and female counterparts. Female patients, however, reported to have a significant increase in anxiety and distress compared to male patients. These findings suggest the pandemic’s diverse and disproportionate impact among patients based on patient characteristics. However, since most patients experienced challenges or psychosocial distress, regardless of their background or profile, incorporating integrative therapies more widely in cancer care would be essential to decrease anxiety/depression-related symptoms and be considered as part of a multidisciplinary approach in the standard of cancer care [20–22]. Furthermore, clinical services, including telehealth, are well received by patients, and increasing the availability of virtual sessions after COVID-19 would be an important addition to cancer care [23].

Our findings suggest that participants of Hispanic origins encountered various challenges, including housing, income, food, education, and healthcare (Table 3). Similar outcomes have been previously reported in the literature [24–26].

**Table 3:** Subgroup analysis for challenges experienced during COVID-19 in Hispanics

Variable	Challenges^a^		*p*-value
	No (*n* = 16)	Yes* (*n* = 15)	
Gender, *n* (%)			0.4331
Male	3 (18.8)	5 (33.3)	
Female	13 (81.2)	10 (66.7)	
Age, *n* (%)			0.2524
34–65	7 (43.8)	3 (20.0)	
≥ 65	9 (56.2)	12 (80.0)	
Race, *n* (%)			1.000
White	14 (87.5)	14 (43.3)	
Black	0 (0.0)	0 (0.0)	
Other	2 (12.5)	1 (6.7)	
Education, *n* (%)			1.000
High school education	2 (12.5)	2 (13.3)	
Any college education	6 (37.5)	6 (40.0)	
Bachelor’s degree or higher education	8 (5.0)	7 (46.7)	
No. of people in the household, *n* (%)			1.000
1	4 (25.0)	4 (26.7)	
> 1	12 (75.0)	11 (73.3)	
Insurance			1.000
Private	8 (50.0)	7 (46.7)	
Other	8 (50.0)	8 (53.3)	

^a^Challenges were defined as any difficulty involving housing, income, food, toiletries, transportation, education, healthcare, or mental health care

*One or more challenges experienced during the COVID-19 pandemic

Furthermore, 60.6% of study participants reported having anxiety and/or depressive symptoms during the pandemic. It is worth mentioning that Hispanics, who constituted 22.8% of our patient population and resemble South Florida’s demographics, received considerably less mental health services compared to others. This is concerning as Hispanic and Latino cancer survivors have been reported to experience significantly more challenges [27, 28].

While patients with cancer have been reported to have amplified stress [29, 30], this could have been exacerbated due to the COVID-19 pandemic [15, 31, 32]. This analysis identified additional significant disparities in receiving access to mental health services based on education status. Notably, patients with less education are getting fewer mental health services.

In this analysis, gender disparities were noted in anxiety and psychological distress, with females experiencing worse outcomes than males. Prior studies have demonstrated an increase in anxiety related to social distancing, physical health symptoms, and fear of COVID-19 [33] in addition to gender differences during stressful experiences [34–36]. It would be important to consider these variations when designing mitigating efforts and evaluating potential gender differences, including telehealth perception and utilization. For example, young female breast cancer survivors expressing psychosocial distress have benefited from educational Zoom meetings [37]. This is particularly relevant since female patients were found to be less comfortable with telehealth compared to males and could use additional integrative and psychosocial services leveraging telehealth.

However, this analysis suggests that socio-demographic characteristics could be associated with reduced readiness to access telehealth. While most of the patients who had at least one virtual appointment reported telehealth as an easy experience and were comfortable using it, several participants seemed uncomfortable with telehealth. Patients over 65 were noted to be more uncomfortable with telehealth. This is consistent with published literature reporting a general satisfaction with telemedicine [38–40] but a comparatively reduced satisfaction among older patients. Possible reasons may be that telehealth does not simulate the traditional patient experience (e.g., no physical examination) and requires them to be trained on technology, eventually compromising the clinician-patient relationship and the availability of resources and referrals for that population [41–43]. We also observed a racial discrepancy concerning the frequency of contact with health care providers, with black patients indicating lower levels of in-person contact with their physicians compared to non-black patients. This is consistent with previous studies that have shown comparable results and have suggested that black individuals have less access to in-person encounters due to limited access to health services, lower levels of income and education among this population, and possible insurance coverage difficulties [44, 45]. Furthermore, our results did not identify any ethnic association, with attitude toward telehealth, likely indicating the widespread access to mobile phones and digital technology. Some studies, however, have suggested that individuals of Hispanic ethnicity were less likely to utilize telehealth during the pandemic [46–48]. Despite the disruption that COVID-19 has caused in cancer care and the increased levels of psychosocial distress among oncology patients [49], promoting telehealth that started during the pandemic as a stable and integrated service in cancer care would increase access to several services to address various aspects of cancer care as well as the often undertreated mental health conditions in oncology. The American Society of Clinical Oncology (ASCO) considers critical the early identification of patients with pre-existing or newly arising anxiety and/or depression symptoms and promotes the use of integrative therapies in addition to the conventional treatment [50, 51]. However, the significant demand for these therapies offered in cancer centers has led to a decrease in their availability and hence, the need to develop new care models using digital strategies and offering a variety of virtual integrative therapies to overcome time delays [52–54].

The findings of this study are significant and contribute to the literature by reporting novel patient-reported outcomes on a promising intervention that could be incorporated into cancer care. The analysis confirmed that oncology patients experienced heightened levels of psychosocial issues exacerbated by the pandemic and that telehealth provides a great resource to integrate in oncology care, particularly for integrative and mental health therapies.

Nevertheless, certain limitations should be noted. First, we have a relatively small sample with a single-center design. Second, patients had the option to leave any item in the questionnaire blank, which limited the power analysis and affected the generalizability of the results.

## Conclusion

This study highlights several important factors related to increased challenges, anxiety, and distress during and after the global pandemic. There has been an increase in mental health issues among patients with cancer and a surge in new care models that incorporate technology to overcome this issue. While this study found that blacks report inferior outcomes, this was not noted in Hispanics. However, Hispanics appear to have less access to mental health services compared to non-Hispanics. This study also highlights disparities based on age and gender that need to be considered to offer a more optimal standard of care in oncology practices.

### Supplementary Information

Below is the link to the electronic supplementary material.

Supplementary file1 (DOCX 34 kb)

## Data Availability

Data is available for investigators who have been approved for access to NCDB files through an application in the ACS website. URL:https://www.facs.org/quality-programs/cancer-programs/national-cancer-database/.
