# Supplementary material for: “You matter”: patients perceptions and disparities about cancer care and telehealth during and after COVID-19 pandemic
Source: Support Care Cancer. 2024 Mar 20;32(4):236. doi: 10.1007/s00520-024-08433-2 (PMC10954847; doi:10.1007/s00520-024-08433-2)
Supplement: Supplementary file 1 — Supplementary file1 (DOCX 34 kb) [file 520_2024_8433_MOESM1_ESM.docx]

| **Variable** | **Challenges** ^a^ | | | **Health ^b^** | | | **Contact with provider ^c^** | | | **Comfort ^d^** | | | **Anxiety/Stress** | | | **Mental/physical health service ^e^** | | |
| --- | --- | --- | --- | --- | --- | --- | --- | --- | --- | --- | --- | --- | --- | --- | --- | --- | --- | --- |
|  | **No**  **(n=75)** | **Yes**  **(n=61)** | **p-value** | **No**  **(n=94)** | **Yes**  **(n=42)** | **p-value** | **No**  **(n=14)** | **Yes**  **(n=122)** | **p-value** | **No**  **(n=35)** | **Yes**  **(n=84)** | **p-value** | **No**  **(n=52)** | **Yes**  **(n=80)** | **p-value** | **No** | **Yes** | **p-value** |
| **Gender, n (%)** |  |  | 0.04† |  |  | 0.298 |  |  | 0.343 |  |  | 0.03† |  |  | 0.04† |  |  | 0.895 |
| Male | 17 (22.7) | 23 (38.3) |  | 25 (26.9) | 15 (35.7) |  | 2 (15.4) | 38 (31.1) |  | 6 (17.1) | 31 (37.4) |  | 21 (40.4) | 19 (24.1) |  | 22 (30.6) | 18 (29.5) |  |
| Female | 58 (77.3) | 37 (61.7) |  | 68 (73.1) | 27 (64.3) |  | 11 (84.6) | 84 (68.9) |  | 29 (82.9) | 52 (62.6) |  | 31 (59.6) | 60 (75.9) |  | 50 (69.4) | 43 (70.5) |  |
| **Age, n (%)** |  |  | 0.768 |  |  | 0.034 |  |  | 0.252 |  |  | 0.04† |  |  | 0.466 |  |  |  |
| 34-65 | 23 (30.7) | 17 (28.3) |  | 33 (35.1) | 7 (17.1) |  | 6 (42.9) | 34 (28.1) |  | 6 (17.1) | 30 (36.1) |  | 14 (26.9) | 26 (32.9) |  | 17 (23.9) | 23 (37.1) | 0.098 |
| ≥65 | 52 (69.3) | 43 (71.7) |  | 61 (64.9) | 34 (82.9) |  | 8 (57.1) | 87 (71.9) |  | 29 (82.9) | 53 (63.9) |  | 38 (73.1) | 53 (67.1) |  | 54 (76.1) | 39 (62.9) |  |
| **Race, n (%)** |  |  | 0.564 |  |  | 0.691 |  |  | 0.01† |  |  | 0.272 |  |  |  |  |  |  |
| White | 254 (72.0) | 48 (28.7) |  | 70 (74.5) | 32 (76.2) |  | 6 (42.9) | 96 (78.7) |  | 23 (65.7) | 64 (76.2) |  | 42 (80.8) | 57 (71.3) |  | 49 (68.1) | 51 (82.3) | 0.005† |
| Black | 15 (20.0) | 8 (13.1) |  | 15 (15.9) | 8 (19.0) |  | 6 (42.9) | 17 (13.9) |  | 10 (28.6) | 13 (15.5) |  | 6 (11.5) | 17 (21.2) |  | 12 (16.6) | 11 (17.7) |  |
| Other | 6 (8.0) | 5 (8.2) |  | 9 (9.5) | 2 (4.8) | 0.254 | 2 (14.3) | 9 (7.4) |  | 2 (5.7) | 7 (8.3) |  | 4 (7.7) | 6 (7.5) |  | 11 (15.3) | 0 (0.0) |  |
| **Ethnicity, n (%)** |  |  | 0.652 |  |  |  |  |  | 1.000 |  |  | 0.124 |  |  | 0.297 |  |  |  |
| Non-Hispanic | 59 (78.7) | 46 (75.4) |  | 70 (74.5) | 35 (83.3) |  | 11 (78.6) | 94 (77.1) |  | 30 (85.7) | 61 (72.6) |  | 43 (82.7) | 60 (75.0) |  | 49 (68.1) | 55 (88.7) | 0.004† |
| Hispanic | 16 (21.3) | 15 (24.6) |  | 24 (25.5) | 7 (16.7) |  | 3 (21.4) | 28 (22.9) |  | 5 (14.3) | 23 (27.4) |  | 9 (17.3) | 20 (25.0) |  | 23 (31.9) | 7 (11.3) |  |
| **Education, n (%)** |  |  | 0.739 |  |  | 0.02† |  |  | 0.765 |  |  | 0.565 |  |  | 0.276 |  |  |  |
| High school or lower | 13 (17.6) | 9 (14.7) |  | 10 (10.7) | 12 (28.6) |  | 2 (14.3) | 20 (16.5) |  | 5 (14.3) | 11 (13.1) |  | 11 (21.6) | 10 (12.5) |  | 15 (21.1) | 7 (11.3) | 0.042† |
| Some college | 28 (37.8) | 27 (44.3) |  | 38 (40.9) | 17 (40.5) |  | 7 (50.0) | 48 (39.7) |  | 17 (48.6) | 33 (39.3) |  | 21 (41.2) | 31 (38.8) |  | 32 (45.1) | 21 (33.9) |  |
| Bachelor’s or higher | 33 (44.6) | 25 (41.0) |  | 45 (48.4) | 13 (30.9) |  | 5 (35.7) | 53 (43.8) |  | 13 (37.1) | 40 (47.6) |  | 19 (37.2) | 39 (48.7) |  | 24 (33.8) | 34 (54.8) |  |
| **No. of people in household, n (%)** |  |  | 0.301 |  |  | 0.276 |  |  | 0.116 |  |  | 0.189 |  |  | 0.315 |  |  | 0.651 |
| 1 | 17 (22.7) | 10 (16.4) |  | 21 (22.3) | 6 (14.3) |  | 5 (35.7) | 22 (18.0) |  | 9 (25.7) | 13 (15.5) |  | 8 (15.4) | 18 (22.5) |  | 15 (20.8) | 11 (17.7) |  |
| >1 | 58 (77.3) | 51 (83.6) |  | 73 (77.7) | 36 (85.7) |  | 9 (64.3) | 100 (82) |  | 26 (74.3) | 17 (84.5) |  | 44 (84.6) | 62 (77.5) |  | 57 (79.2) | 51 (82.3) |  |
| **Insurance, n (%)** |  |  | 0.449 |  |  | 0.068 |  |  | 0.662 |  |  | 0.061 |  |  | 0.322 |  |  | 0.166 |
| Private | 49 (65.3) | 36 (59.0) |  | 54 (57.5) | 31 (73.8) |  | 8 (57.1) | 77 (63.1) |  | 9 (25.7) | 37 (44.1) |  | 17 (67.3) | 47 (58.8) |  | 49 (68.1) | 35 (56.5) |  |
| Other | 26 (34.7) | 25 (41.0) |  | 40 (42.5) | 11 (26.2) |  | 6 (42.9) | 45 (36.9) |  | 26 (74.3) | 47 (55.9) |  | 35 (32.7) | 33 (41.2) |  | 23 (31.9) | 27 (43.5) |  |

**Supplementary Material**

Table 1. Univariate analysis for the different events of interest

†Statistically significant

^a^ Challenges were defined as any difficulty involving housing, income, food, toiletries, transportation, education, healthcare, mental health care or others.

^b^ Participants perceptions regarding the improvement of their overall health (physical and mental) since the beginning of the COVID-19 pandemic.

^c^ Refers to participants’ perceptions regarding increase contact with their provider since the onset of the COVID-19 pandemic.

^d^ Refers to whether the participants experienced a sense of comfort using telehealth services during COVID-19 pandemic.

^e^ Refers to the increased utilization of mental and physical health services during the COVID-19 pandemic

**Supplementary Material**

**Table 2**. **21-item “You matter: Covid-19 Mental Health Impact Survey”**

| **YOU MATTER : COVID 19 MENTAL HEALTH IMPACT SURVEY**  MODIFIED FOR: ‘The Impact of COVID 19 on Social and Mental Health, and Access to care for Patients with Cancer at a Comprehensive Academic Cancer Center.’ | |
| --- | --- |
| **DEMOGRAPHICS** | |
| 1. What is your age? | - 18 - 24 - 25 - 34 - 35 – 44 - 45 - 54 - 55 – 64 - >65 |
| 1. Are you of Hispanic or Latino? | - No - Yes - Unknown |
| 1. If Hispanic, please specify. | - Cuban - Puerto Rican - Ecuadorian - Dominican - Mexican - Cape Verdean - Brazilian - Unknown - Not applicable |
| 1. What is your race? | - White - Black/African American - Asian - American Indian/Alaskan Native Asian - Native Hawaiian/Other Pacific Islander - Unknown - Other (Please Specify) |
| 1. Which of the following BEST captures your gender identification? | - Male - Female - Other - I don’t wish to answer |
| 1. What is the highest level of education you completed? | - Less than high school - High school or equivalent. (e.g., GED) - Some college, including associate degree or trade school - Bachelor’s degree or higher |

| **SOCIAL HEALTH** | |
| --- | --- |
| 1. Which option best describes your current health insurance coverage? | - No Insurance/Self-pay - Medicaid/Medicare - Private insurance (e.g., Aetna, Cigna) - Other |
| 1. Did your health insurance coverage change during the pandemic? | - Yes, I lost coverage - No, it did not change my coverage - Yes, I gained coverage |
| 1. How many people live in your household including yourself? | - 1 - 2 - 3 - 4 - 5+ |
| 1. Please select areas where you experienced challenges because of COVID-19 (between April – June 2020) | - Housing - Income/benefits Employment - Food - Toiletries and clean clothes - Transportation - Education/online education - Healthcare - Mental health care - Other (please specify) ___________ |
| 1. Since April 2020, would you say the level of stress in your life has increased, decreased, or has it stayed about the same? | - Decreased - Stayed the same - Increased |
| 1. **Since April 2020, would you say each of the following aspects of your health has gotten better, worse, or stayed about the same?** | |
| **Overall health** | 1. Much worse 2. Somewhat worse 3. About the same 4. Somewhat better 5. Much better |
| **Physical health** | 1. Much worse 2. Somewhat worse 3. About the same 4. Somewhat better 5. Much better |
| **Mental health** | 1. Much worse 2. Somewhat worse 3. About the same 4. Somewhat better 5. Much better |

| **TELEHEALTH ATTITUDES** | |
| --- | --- |
| 1. Have you participated in telehealth appointments since April 2020? | - Yes - No |
| 1. If yes, please select the mode of communication (select all that apply) | - Telephone - Video conference on a cell phone - Video conference on an iPad, tablet or computer - E-mail - Text message |
| 1. Please select your experience with telehealth | - It was easy and effective - It was difficult and hard to use - It was impossible because I don’t have a phone or computer |
| 1. Since April 2020, please select the option that BEST describes your contact with providers | - Less contact with providers since COVID-19 - Same contact with providers since COVID-19 - More contact with providers since COVID-19 |
| 1. What is preventing you from using telehealth services? (Select all that apply) | - Don’t have a phone - Not enough minutes, data support, or room on phone for apps - Don't have internet access - Not offered telehealth services - Not comfortable - Don’t have a computer - Does not apply |

| 1. Please rate your comfort level with telehealth | - Very uncomfortable - Uncomfortable - Neutral - Comfortable |
| --- | --- |

| **MENTAL HEALTH** | |
| --- | --- |
| 1. Have you received in-person mental health or Physical Health services since April 2020? | - No - Yes, mental health services - Yes, Physical health services - Yes, both mental health and physical health services |
| 1. Please select the option that best describes your mental health since April 2020. | - Have not experienced anxiety, stress or distress as a result of COVID-19 - Slight increase in anxiety, stress or experience of distress as a result of COVID-19 - Moderate increase in anxiety, stress or experience of distress as a result of COVID-19 - Significant increase in anxiety, stress or experience of distress as a result of COVID-19 - Have not experienced anxiety, stress or distress as a result of COVID-19 |
| 1. Please select the option that BEST reflects your experience with receiving mental health or substance use services (e.g., counseling, therapy, case management). | - No difficulty in receiving services - Some difficulty receiving services (e.g., because of social distancing measures, transportation issues, less support) - Significant difficulty due to not being able to connect with treatment providers - Does not apply - Other (please specify): |
